# Negative regulation of diminutive cancer regulator through differentiation and microRNA pathway components in Drosophila cells

**DOI:** 10.3906/biy-2012-4

**Published:** 2021-04-20

**Authors:** Sumira MALIK

**Affiliations:** 1 Amity institute of Biotechnology, Amity University Jharkhand, Ranchi, Jharkhand India; 2 School of Biological Sciences and Technology, Chonnam National University the Republic of Korea

**Keywords:** Bam, Brat, AGO1, microRNA, dMyc

## Abstract

*Drosophila* model is intensively studied for the development of cancer. The diminutive (dMyc), a homolog of the human MYC gene, is responsible for cell- apoptosis and its upregulation is responsible for determining the fate of cancerous growth in humans and *Drosophila* model. This work implores the requirement of dMyc and its expression as one of the major regulator of cancer with other proteins and repression of dMyc mRNA in *Drosophila* S2 cells. Here we report protein complex of Argonaute 1 (AGO1), Bag of marbles (Bam), and Brain tumor (Brat) proteins and not the individual factor of this complex repression of dMyc mRNA in *Drosophila* Schneider 2 cells and promote differentiation in cystoblast of *Drosophila* ovary. These results exhibit the significant role of this complex, including master differentiation factor Bam with other various differentiation factor Brat and microRNA pathway component AGO1, which may negatively regulate dMyc mRNA and so the dMyc protein.

## 1. Introduction

In the *Drosophila *ovary, the balance between symmetric and asymmetric divisions is tightly regulated to control and produce appropriate amounts of proliferating stem cells and differentiated cells. The mechanisms that control the balance between stem cell self-renewal and differentiation depend on the coordinated regulation of complex transcriptional and posttranscriptional hierarchies (Fuller and Spradling, 2007; Di Giacomo et al., 2017). The antagonistic relationship mediates the balance between germ-line stem cell (GSC) self-renewal and differentiation in Drosophila ovaries between the GSC self-renewal factors and expression of a key differentiation factor Bag of Marbles (Bam) in *Drosophila *germline.

*Drosophila* dMyc is highly expressed in the differentiating cystoblast and dividing cysts of *Drosophila* ovary. The differential expression of dMyc triggers competitive interactions among stem cells and differentiating daughters promoting the differentiation event (Rhiner et al., 2009; Li et al., 2012). dMyc is responsible for cellular growth and other functions, and its repression is known to be regulated through the microRNA (Johnston et al., 1997; Daneshwar et al., 2013). Another differentiation factor Brain tumor (Brat), was also known to regulate its expression of *dMyc* in cystoblast individually (Harris et al., 2011; Daneshwar et al., 2013). Bam was the first intrinsic responsible for CB differentiation (McKearin, and Spradling, 1990; Ohlstein et al., 2000). Bam and benign gonial cell neoplasm (bgcn) were required in the repression of Nanos protein expression in differentiated germ cells through 3’UTR of its mRNA with an unknown mechanism that indicates the presence of complexes in mRNA repression (Li et al., 2009; Kim et al., 2010). Argonaute 1 (AGO 1), the member of the RISC pathway, has an important role in the microRNA pathway and degradation of target mRNAs through miRNA sites (Antic et al., 2015).

This study reveals that AGO1, Bam, and Brat complex rather than individual protein is required for binding and repression of reporter harboring the 3’UTR of *dMyc *mRNA in S2 cells. The luciferase reporter and RNA-protein immunoprecipitation assays in S2 cells signify the collaborative repression of each of *dMyc* mRNA through Bam, Brat and AGO 1. These findings highlight role of this complex rather than individual in the repression of key stem cell maintenance factors Myc. Hence, the multiple complexes of Bam, Brat, and AGO1 rather than single factors might be required in the repression of dMyc expression in cystoblast.

## 2. Materials and methods

### 2.1. Yeast two-hybrid X-gal and assay

The full-length sequences of Bam, Brat and, AGO1 full length were cloned into various sites of pLex A and pACT2 (Clontech). These combinations of plasmids expressing the Lex A-fused protein and GAD-fused protein were cotransformed into the yeast strain YPH500 (MAT ade 2, his3, leu 2, lys2, trp1, ura3) harboring the pSH18-34 plasmid (lexAop-LacZ reporter) by the standard lithium acetate method (Ito et al., 1983; Gyuris et al., 1993). The independent transformants were patched onto glucose plates containing X-gal for 24–48 h and followed by liquid assay and reported as beta-galactosidase units.

### 2.2. Coimmunoprecipitation assay and siRNA silencing

The expression vectors and their vectors were cloned and were transiently transfected with the appropriate set of expression plasmids using the dimethyldioctadecyl-ammonium bromide (DDAB) method (Malik et al., 2019). The (*Drosophila* Schneider’s 2 cells). According to Han (1996), S2 cells were maintained in Shields and Sang M3 insect medium (Sigma) supplemented with 10% insect medium supplement (Sigma) and antibiotics in humidified atmosphere at 25 °C and then the cells were transiently transfected with the appropriate set of expression plasmids using the DDAB method. For the coimmunoprecipitation assay, transfected cells were harvested 72 h after transfection and the cells were washed in phosphate-buffered saline and lysed with radioimmune precipitation assay buffer (50 mM Tris-HCl, pH 8.0, 150 mM NaCl, 1% Nonidet P-40, 5mM EDTA, and 1 mM phenylmethylsulfonyl fluoride; ELPIS Biotech. Inc., Seo-gu, the Republic of Korea) containing protease inhibitor mixture. The lysates were clarified by centrifugation at 13,000 rpm (Eppendorf centrifuge) for 10 min at 4 °C. The cleared extracts (3 mg) were mixed with 40 uL of anti-FLAG M2-conjugated agarose beads (Sigma), 10 uL of anti-HA antibody was added to sepharose beads to conjugate the anti-HA antibody (mouse) and rotated at 4 °C overnight. The beads were precipitated by Eppendorf centrifugation and washed three times with 20 mM HEPES (pH 7.7), 150 mM NaCl, 2.5 mM MgCl2, 0.05% Nonidet P-40, 10% glycerol, and 1 mM dithiothreitol containing protease inhibitor mixture. The bound proteins were eluted using 0.1 mM glycineacetate (pH 3.0), and the eluates were precipitated with the trichloroacetic acid. The pellets were resuspended in 2X SDS loading buffer, and Western blot analysis was performed using anti-HA (rat) (Roche Applied Science), anti-Bam (DSHB) Developmental studies of Hybridoma Bank (mouse), anti-Pum 1637 (gift from P. M. Macdonald), anti-Myc (rabbit) (Cell Signaling, Danvers, MA, USA), and anti-FLAG (M2; Sigma) (mouse) antibodies in 1:3000 dilution ratio in 5 % skimmed milk. The HRP-conjugated goat antirabbit secondary antibodies, HRP-conjugated goat antimouse secondary antibodies, HRP-conjugated goat antirabbit secondary antibodies, HRP-conjugated goat antirat secondary antibodies were used in 1:5000 dilution ratio in 5 % skimmed milk and ECL western blotting detection kit (Amersham Bioscience) was used for protein interaction. For silencing of Brat protein, synthetic oligonucleotide duplexed Brat siRNA oligomers were diluted RNase-free environment in DEPC water as working stocks of 20 pmol/uL, according to the (Bioneer Corporation, Daejeon, the Republic of Korea) company’s protocol and were transfected with the given set of plasmid expression vectors in Drosophila S2 cells.

### 2.3. Luciferase reporter assay 

For the construction of reporter plasmids dMyc3’utr full length and its derivatives *dMyc* 1-200 3’utr, according to Han K. (1996) DDAB method*, dMyc* 201-400 3’utr and *dMyc*-401-674 3’utr were cloned and transfected with combinations of expression vectors were co-transfected using. S2 cells were plated into a 24-well plate, and the cells were harvested and assayed 72 h posttransfection for luciferase activity and normalized with b-gal activity. The results were obtained from triplicate samples, and data are representative of a minimum of three to five independent experiments.

### 2.4. RNA-protein immunoprecipitation assay

The expression vectors were cloned and were transiently transfected with the appropriate set of expression plasmids using the DDAB method. For the coimmunoprecipitation assay, transfected cells were harvested 72 h after transfection, and the protocol was followed (Neumüller et al., 2008; Malik et al., 2019).

### 2.5. Statistical analysis

All experiments were performed at least three times. Data calculation and statistical analysis were performed using GraphPad Prism software 6.0 software. 

## 3. Results

### 3.1. The binary complexes show direct and robust interaction of Brat-Bam, Brat-AGO 1, and no interaction of Bam and AGO1 proteins in Yeast and coimmunoprecipitation assay in S2 cells

To analyze the binary complex among Bam, Brat, and AGO 1 protein, yeast two-hybrid assay was performed, either Bam full length, AGO 1 full length and Brat derivatives, were fused to either the Lex A DNA-binding domain (DBD) or the pACT2 transcriptional activation domain (TAD) in various combinations (Figure 1a). In the yeast two-hybrid assay, X-GAL patching on glucose plates was performed, followed by liquid assay. A stronger binary interaction with higher beta-galactosidase units was detected among Brat with AGO 1 full length and Brat interaction with Bam. However, Bam failed to interact with AGO 1 directly, and it indicates the possibility of formation of this complex through Brat, which interacts directly with AGO1 and Bam, both mediating the Bam, Brat, and AGO 1 multiple complexes as summarized in Figure 1a. Furthermore, the coimmunoprecipitation assays confirm the presence of Brat, AGO1, and Bam protein complex in S2 cells (Figure 1b) (Lane 5) where Brat acts as a bridging protein. Upon endogenous depletion of brat through siRNA silencing AGO1 and Bam fails to precipitate which confirms that for the formation of Bam, AGO1, and Brat complex, Brat is required (Figure 1b) (Lane 4).

**Figure 1a:**
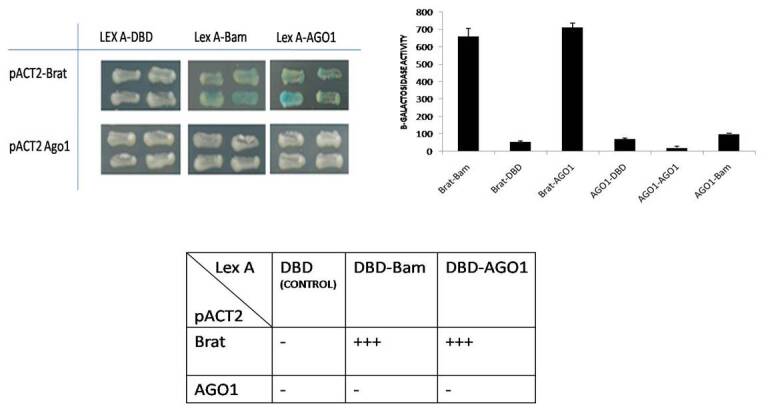
A yeast two-hybrid assay on X-Gal plates and B-galactosidase assays. The different combinations of Bam, AGO1, and Brat full length containing GAD and Lex A DBD constructs were examined in the form of X-Gal patching assay andβ-galactosidase activity with YPH 500 strain of yeast. Colonies from the X-Gal plate were transferred to Liquid β-galactosidase assays were carried out for transformants.

**Figure 1b:**
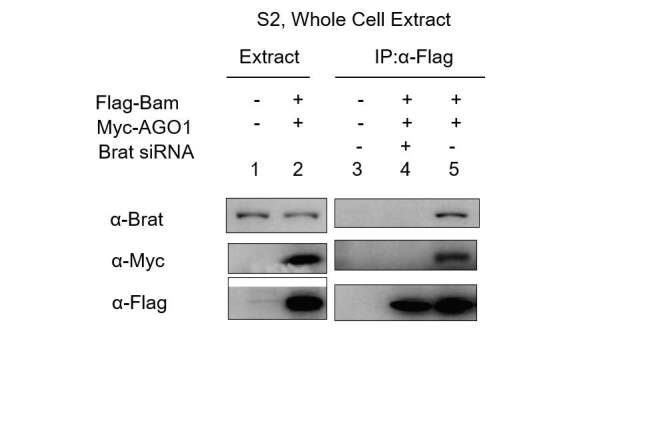
S2 cells showing coimmunoprecipitation multiple protein complex of Bam, AGO 1, and Brat through presence of important factor Brat; Bam, AGO 1, and Brat forms a multiple Protein complex (Lane 5) in lysates of S2 cells expressing Flag-tagged Bam, Myctagged AGO 1 after being precipitated with anti-Flag conjugated agarose beads and Bam and Ago 1 fails to be present in the complex in absence of Brat on endogenous depletion of Brat using Brat siRNA (Lane 4).

### 3.2. Bam, Brat, and AGO1 proteins cooperatively and not individually form multiple protein complexes and represses expression of dMyc 3’UTR full-length luciferase reporter in Drosophila S2 cells

**Figure 2:**
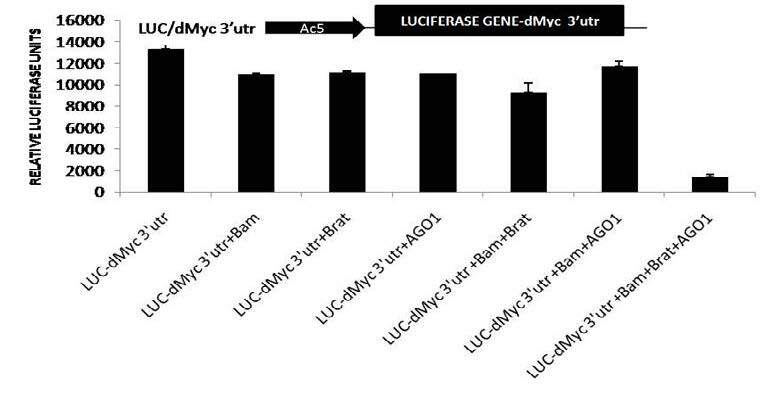
A d iagram of a luciferase reporter containing (Luc) coding sequence with Actin promoter (black arrow) and dMyc 3’UTR (black box). Drosophila S2 cells transfected with Brat, Bam, and AGO1 expression vectors, luciferase reporter, in several combinations. Luciferase activities were measured, and the mean ± SD values were obtained from at least three independent experiments performed in triplicate.

Luciferase reporter assay revealed that the Bam, Brat, and AGO1 factors failed to repress dMyc 3’UTR independently but surprisingly, Bam collaboratively showed remarkable repression with Brat and AGO1 proteins, which strikingly depicts that Bam, Brat, and AGO1 proteins only collectively show the function of dMyc 3’UTR repression as shown Figure 2.

### 3.3. A multiprotein complex of Brat, AGO1, and Bam represses expression of luciferase reporter containing microRNA like sequences in 1-200 bp region of dMyc 3’UTR

**Figure 3:**
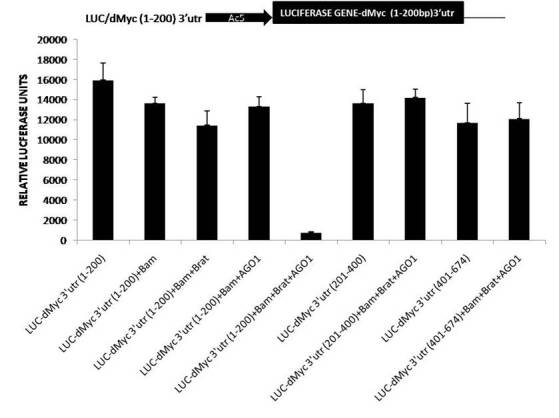
(Upper) Schematic diagram of a reporter containing luciferase (luc) sequence with Actin promoter (black arrow Myc 3 ’UTR [1-200] bp [(black box). (Lower) Drosophila S2 cells transfected with Brat, Bam, and AGO1 expression vectors, Luciferase reporter, in several combinations.

dMyc 3’UTR was studied in three parts of (1–200 bp) region, (201–400 bp) region, and (401–674 bp) region. A previous study highlighted the role of [401–674 bp region] containing specific microRNA sites and mediated repression in *Drosophila melanogaster *(15). Collectively these results corroborate that Bam, Brat, and AGO1 proteins form a multiple complex and functions in the repression of dMyc 3’UTR (1–200 bp) that contains microRNA like sites. In contrast, Bam, Brat, and AGO1 multiple complexes failed to show effective repression in dMyc 3’UTR (201–400 bp) and dMyc (401–674bp) 3’utr repression, suggesting that Bam require Brat to facilitate the AGO1 miRNA pathway mediated translational repression of microRNA like sites containing 1–200 bp region in dMyc 3’UTR of *dMyc *mRNA regulation in cystoblast as explained in Figure 3.

### 3.4. Brat, Bam, Bgcn, Mei-P-26, and AGO 1 precipitates show an association of Brat, Bam, Bgcn, Mei-P-26, and AGO 1 protein with nos mRNA and dMyc mRNA in Drosophila S2 cells

**Figure 4:**
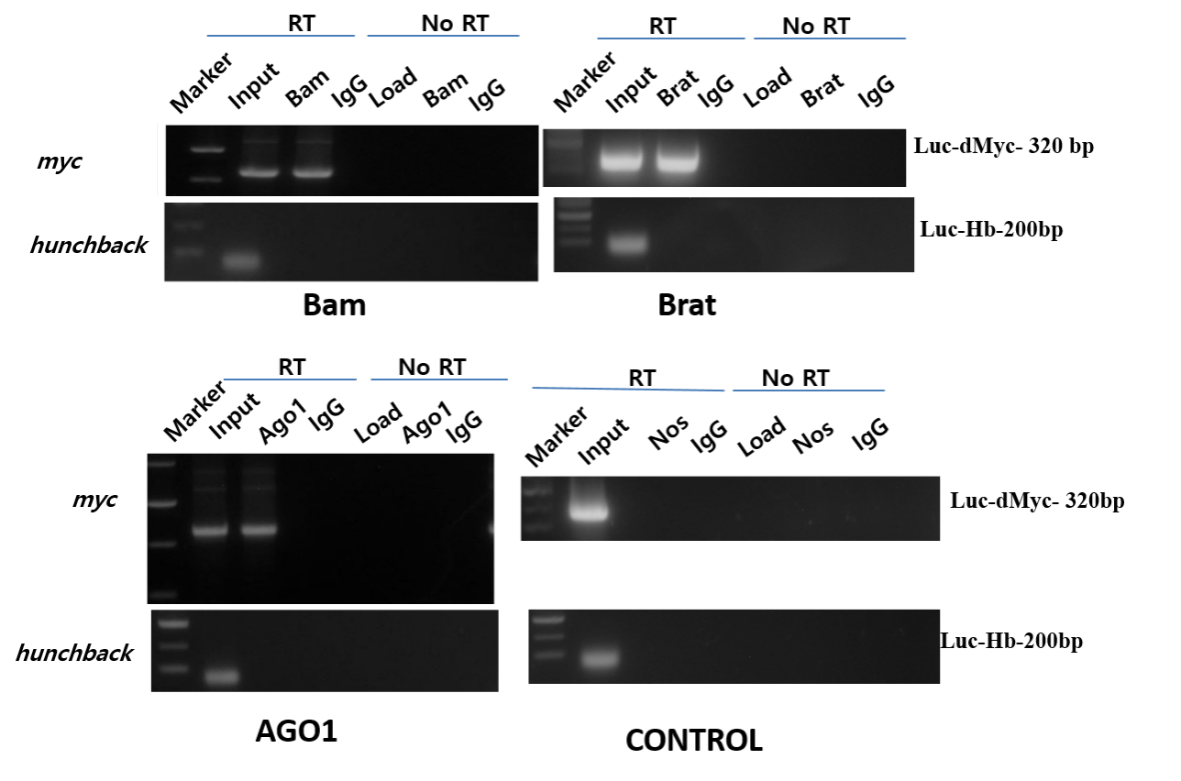
The immunoprecipitates of Brat, Bam, and AGO 1 protein shows the presence of dmyc mRNA where control Nos failed to show the presence of dmyc mRNA in Drosophila S2 cells.

A protein complex of Bam-Brat-AGO 1 repressed *dMyc*3’UTR (Figure 3). To confirm whether this complex binds to *dMyc*3’UTR, the RNAs were immunoprecipitated with Bam, Brat, and AGO 1 antibody, then reverse transcription followed by PCR using primers corresponding to *dMyc *mRNA. In contrast, Nos immunoprecipitates acted as a control and failed to show an association of *dMyc *mRNA Figure 4. Therefore, this confirms that Bam-Brat-AGO 1 and each constituent of this complex binds to *dMyc* mRNA. And mediate translational repression in *Drosophila* S2 cells through microRNA sites in the region of 1–200 base pairs.

## 4. Discussion 

Here, we present the multiple protein complexes of Bam, Brat, and AGO1, in which the Brat protein cooperates with both Bam and AGO1, which are indirectly interacting proteins in the complex of Brat, Bam, and AGO1 protein complex. Together this coregulates the repression of self-renewal mRNA through miRNA in 1–200bp region as explained in Figures 2–4. However, the other constructs from region 201–674 bp showed no repression through this, complex as explained in Figure 3. The previous studies showed that in *Drosophila *embryos Brat and AGO1 that were precipitated together were found functionally active in maintenance of differentiation of cystocytes in flies (19). Figures 1a and 1b (Lane 5) of yeast 2 hybrid assay and coimmunoprecipitation assay confirms the presence of Brat, Bam, and AGO complex. However, upon endogenous depletion of Brat, using Brat siRNA in S2 cells Bam and AGO1 failed the precipitation in S2 cells Figure 1b (Lane 4). Thus in this data, we confirmed that Brat acts as bridging protein among Bam and AGO1 and due to the presence of direct interaction among Brat-Bam, Brat-AGO1 proteins a stable multiple protein complexes. Bam, Brat, and AGO1 might get stabilized and brought AGO1 a microRNA pathway component to the microRNA sites in 1–200 bp region of dMyc 3’utr of dMyc mRNA making complex functionally active in CBs or differentiating cysts. According to Malik (2020), the presence of microRNA sites and the binding of Bam, Brat, and Mei-P26 factors on dMyc mRNA through microRNA sites has been reported in S2 cells.

**Figure 5:**
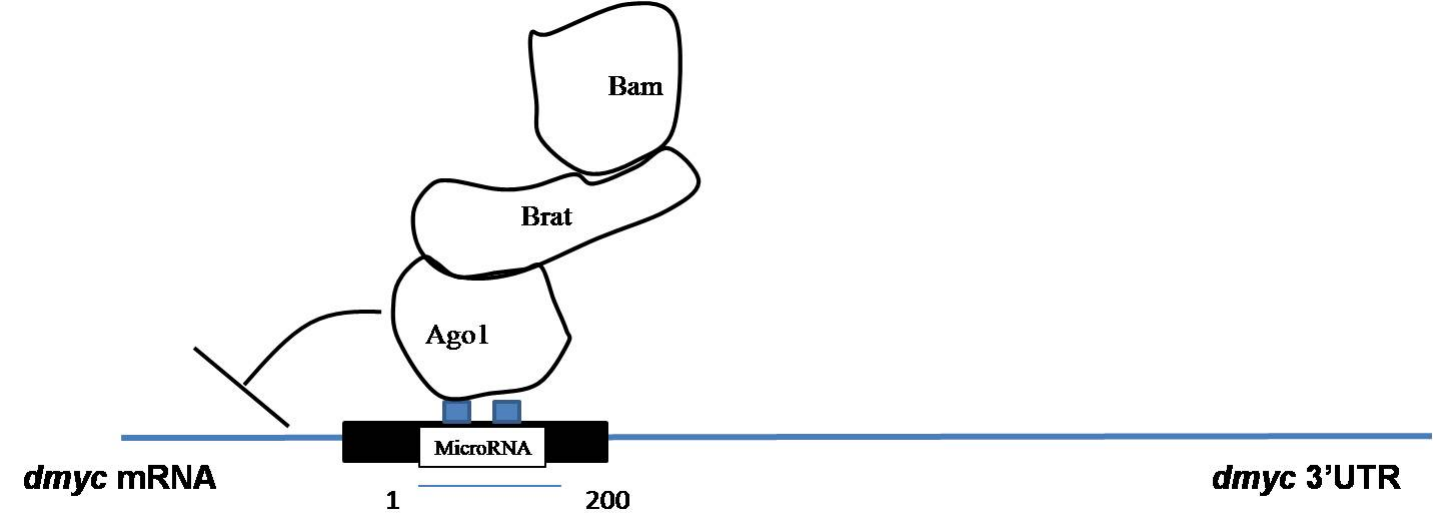
A model depicting the possible mechanism for dMyc repression in cystoblast. A model for the Bam, Brat, and AGO1 multiple protein complexes mediated translational repression of dMyc mRNA through the miRNA pathway that might negatively regulate selfrenewal mRNA dMyc expression, in cystoblast (CB) by binding to microRNA like sequences of dMyc 3’UTR highlighted in the 1–200 bp region.

These results are in accordance with previous genetics studies and biochemical studies, which also showed the combined function of Brat and AGO1 and Bam and Brat independently in GSC maintenance and cystocytes accordingly (Pierce et al., 2004; Malik et al., 2017). Brat, a differentiation promoter protein, shows higher expression in CB than GSC. In this analysis, as shown in Figure 2, luciferase reporter assays confirmed the presence of the multiple protein complexes of Bam, Brat, and AGO1 proteins, and their translational repression of *dMyc* mRNA. However, when they were transfected individually and in combination of two proteins to further investigate and confirm their role as independent entity, no repression was found whereas in combinations of Bam, Brat, and AGO1 through 1–200 bp region in 3’UTR of *dMyc* m RNA, the repression of luciferase activity was observed as explained in Figures 2 and 3. This data could be supported by the previous analysis which showed that Brat regulates levels of dMyc. In the CB, domain-containing Brat, functions with AGO1, a microRNA RISC component and differentiation factor as Bam and mediated silencing of *dMyc* mRNA through positive regulation of miRNA pathway and mediated translation repression of dMyc mRNA in differentiating cystoblast. Furthermore, the immunoprecipitates of Bam, Brat, and AGO1 in whole-cell extracts of *Drosophila* cells with dMyc mRNA confirmed the presence of Bam, Brat, and AGO1 proteins bonded with dMyc mRNA in form Protein-RNA complexes through RNA immunoprecipitations assays as shown in Figure 4. Therefore, it is concluded that multiple protein complexes of Bam, Brat, and AGO1 complex in CBs and negatively regulate *dMyc* mRNA by binding with microRNA-like sites in 1–200 region of dMyc 3’UTR, promoting germline differentiation explained by a model predicting the possible mechanism for dMyc repression in cystoblast in Figure 5.

## Funding

This work was supported by the Bio and Medical Technology Development Program [grant numbers. NRF-2015R1A2A2A01004803, NRF-2018R1D1A1B07049100]; and National Research Foundation (NRF) [grant number 2017M3A9D8048708].

## Ethical clearance

Clearance for data used and obtained during doctoral program was obtained from School of Biological Sciences, Lab of Stem Cell Biology, Chonnam National University, Kwangju, Republic of Korea Research Committee.
